# Efficacy and safety of cladribine, low-dose cytarabine and venetoclax in relapsed/refractory acute myeloid leukemia: results of a pilot study

**DOI:** 10.1038/s41408-024-00982-3

**Published:** 2024-01-18

**Authors:** Yan-Yan Li, Shuai-Shuai Ge, Yuan-Hong Huang, Ming-Zhu Xu, Chao-Ling Wan, Kai-Wen Tan, Tao Tao, Hai-Xia Zhou, Sheng-Li Xue, Hai-Ping Dai

**Affiliations:** 1 National Clinical Research Center for Hematologic Diseases, Jiangsu Institute of Hematology, The First Affiliated Hospital of Soochow University, Suzhou, China; 2 Institute of Blood and Marrow Transplantation, Collaborative Innovation Center of Hematology, Soochow University, Suzhou, China; 3 Department of Respiratory and Critical Medicine, The Affiliated Infectious Diseases Hospital of Soochow University, Suzhou, China

**Keywords:** Acute myeloid leukaemia, Combination drug therapy

Dear Editor

The 3-year overall survival for patients with primary refractory or relapsed acute myeloid leukemia (R/R AML) is only approximately 10% [1]. Allogeneic hematopoietic stem cell transplantation (allo-HSCT) is a curative treatment for R/R AML, but has a high post-transplant relapse in patients who received transplant with active disease. Reduction of pre-transplant leukemia burden by salvage therapy can reduce post-transplant relapse [2]. Conventional salvage chemotherapy often has a low response rate but high organ toxicities [3]. Therefore, exploring novel salvage regimens to bridge allo-HSCT may help improve the prognosis of R/R AML.

Venetoclax plus hypomethylating agents (HMA) or low-dose cytarabine (LDAC) is recommended for newly diagnosed older or unfit AML patients. However, the efficacy of a venetoclax-based low-intensity regimen for R/R AML was unsatisfactory, with a response rate of 21% and a median survival of 3.0 months [4]. Venetoclax resistance is closely related to monocytic disease progression. In a recent study, cladribine (CLAD) is a potent DNA polymerase and ribonucleotide reductase inhibitor, selectively killed monocytic leukemia stem cells (LSCs) via reliance on purine metabolism. CLAD and venetoclax have demonstrated synergetic efficacy for AML due to the eradication of multiple LSCs [5, 6]. CLAD also synergizes with cytarabine [5]. CLAD and LDAC alternated with decitabine showed a response rate of 64% in newly diagnosed (ND) AML [7]. A phase II study demonstrated a composite complete remission (CRc) rate of 93% with CLAD, LDAC and venetoclax in ND AML [8]. Because the efficacy of CLAD, LDAC and venetoclax in R/R AML is unknown, we conducted a phase 2 study (NCT05190549) utilizing the CAV regimen (CLAD, LDAC and venetoclax) for R/R AML.

This trial enrolled R/R AML patients aged 16–70 years, who have adequate organ functions, ECOG score ≤ 1 and an expected survival of ≥3 months. Diagnosis, risk stratification, relapse and treatment response are defined according to the 2017 ELN recommendations [9]. Refractory was considered as: (1) failure to achieve CR/CRi after two courses of intensive induction treatment. (2) failure to achieve CR/CRi after one course of induction therapy, with a reduction of <50% in the number of blasts or residual blasts >15% [9, 10]. Written informed consent was obtained according to the Declaration of Helsinki.

Patients received intravenous CLAD 5 mg/m^2^ for 5 days, subcutaneous cytarabine 20 mg every 12 h for 10 days, and oral venetoclax began at 100 mg on day 1 and increased stepwise over 3 days to reach the target dose of 400 mg for 21 days. Venetoclax treatment may be temporarily interrupted in the event of neutropenic infection. Responders to CAV may receive allo-HSCT or consolidation therapy according to whether a suitable donor was available. Nonresponders can receive a second cycle of CAV or change to other regimens at the physician’s discretion. Bone marrow (BM) evaluation was performed within 4 weeks after the completion of CAV, prior to the start of new anti-leukemia treatment or before conditioning chemotherapy for allo-HSCT. Measurable residual disease (MRD) was detected by flow cytometry. Overall response rate (ORR) was calculated as the sum of CR, CRi, MLFS and PR. CRc was calculated as the sum of CR and CRi. Overall survival (OS) was defined as the time from the initiation of CAV to death from any cause or the last follow-up. EFS was defined as the time from the initiation of CAV to treatment failure, relapse, death from any cause or the last follow-up. Adverse events (AEs) were evaluated according to CTCAE (V5.0) [11]. Statistical methods were described in Supplementary materials.

Between October 1, 2021 and January 18, 2023, 30 R/R AML patients were enrolled, including 16 males and 14 females. The median age at enrollment was 39.5 (range, 16–68) years. Two patients had AML secondary to MDS and 28 were de novo AML. Eleven patients had M4/M5 FAB subtype. The mutational landscape of the patients was shown in Supplementary Fig. 1. The number of patients in favorable, intermediate and adverse risk groups were 14, 5 and 11, respectively. There were 10 primary refractory AML and 20 relapsed AML (Table 1). Twenty-four patients were refractory to standard 7 + 3 or relapsed after consolidation with intermediate dose cytarabine. Thirteen patients received treatment with venetoclax previously. The median number of prior lines of therapy before enrollment was 2 (range, 1–4) (Supplementary Table 1). Twenty-two patients received 1 cycle and 8 patients received 2 cycles of CAV. Eleven (36.7%) patients received CAV as the first salvage treatment. Treatments after CAV were shown in Supplementary Table 2.

**Table 1:** Patient baseline characteristics and response outcomes.

Characteristic	All patients (*n* = 30)	Refractory AML (*n* = 10)	Relapsed AML (*n* = 20)	*P*
*Age, years*	39.5 (16–68)	50 (22–68)	38.5 (16–67)	0.441
16–60, No (%)	26 (86.7)	8 (80.0)	18 (90.0)	
>60, No (%)	4 (13.3)	2 (20.0)	2 (10.0)	
*Gender, No (%)*				0.442
Male	16 (53.3)	4 (40.0)	12 (60.0)	
Female	14 (46.7)	6 (60.0)	8 (40.0)	
WBC (×10^9^/L)	2.5 (0.4–37.0)	1.0 (0.4–14.4)	3.3 (0.5–37.0)	**0.009**
Platelets (×10^9^/L)	40.5 (6–314)	56.5 (6–314)	31 (10–198)	0.158
Hemoglobin, g/L	75.3 (53–153)	58.5 (53–78)	78.5 (59–153)	**<0.001**
Bone marrow blasts	39.8 (6.0–99.0)	37.3 (8.0–79.5)	42.5 (6–99)	0.673
<50%	19 (63.3)	7 (70.0)	12 (60.0)	
≥50%	11 (36.7)	3 (30.0)	8 (40.0)	
Secondary AML	2 (6.7)	1 (10.0)	1 (5.0)	1.000
*Risk stratification at diagnosis (2017 ELN), No (%)*				0.111
Favorable	14 (46.7)	2 (20.0)	12 (60.0)	
Intermediate	5 (16.7)	2 (20.0)	3 (15.0)	
Adverse	11 (36.7)	6 (60.0)	5 (25.0)	
*Prior exposure to VEN, No (%)*				1.000
Yes	13 (43.3)	4 (40.0)	9 (45.0)	
No	17 (56.7)	6 (60.0)	11 (55.0)	
*FAB subtype, No (%)*				0.702
M4/M5	11 (36.7)	3 (30.0)	8 (40.0)	
Not M4/M5	19 (63.3)	7 (70.0)	12 (60.0)	
Bridge to Allo-HSCT, No (%)	18 (60.0)	5 (50.0)	13 (65.0)	0.693
*Cytogenetics*				
t(v;11q23)/KMT2A-rearranged	6 (20.0)	3 (30.0)	3 (15.0)	0.372
Monosomy 7	1 (3.3)	1 (10.0)	0 (0)	0.333
Complex karyotype	1 (3.3)	1 (10.0)	0 (0)	0.333
*Mutations, No (%)*				
*RAS*	10 (40.0)	3 (30.0)	7 (35.0)	1.000
*NRAS*	9 (30.0)	3 (30.0)	6 (30.0)	1.000
*KRAS*	3 (10.0)	1 (10.0)	2 (10.0)	1.000
*CEBPA*	7 (23.3)	1 (10.0)	6 (30.0)	0.372
*FLT3*	7 (23.3)	1 (10.0)	6 (30.0)	0.372
*FLT3*-ITD	4 (13.3)	0 (0)	4 (20.0)	0.272
AR < 0.5	3 (10.0)	0 (0)	3 (15.0)	
AR ≥ 0.5	1 (3.3)	0 (0)	1 (5.0)	
*FLT3*-TKD	4 (13.3)	1 (10.0)	3 (15.0)	1.000
*WT1*	6 (20.0)	1 (10.0)	5 (25.0)	0.633
*KIT*	5 (16.7)	0 (0)	5 (25.0)	0.140
*RUNX1*	4 (13.3)	1 (10.0)	3 (15.0)	1.000
*NPM1*	3 (10.0)	1 (10.0)	2 (10.0)	1.000
*TET2*	3 (10.0)	1 (10.0)	2 (10.0)	1.000
*DNMT3A*	2 (6.7)	1 (10.0)	1 (5.0)	1.000
*PTPN11*	2 (6.7)	1 (10.0)	1 (5.0)	1.000
*SRSF2*	2 (6.7)	0 (0)	2 (10.0)	0.540
*U2AF1*	2 (6.7)	1 (10.0)	1 (5.0)	1.000
*Response outcomes*				
ORR, No. (% [95% CI])	21 (70.0 [52.1–83.3])	6 (60.0 [31.3–83.2])	15 (75.0 [53.1–88.8])	0.673
CRc, No. (% [95% CI])	8 (26.7 [14.2–44.5])	3 (30.0 [10.8–60.3])	5 (25.0 [11.2–46.9])	1.000
CR, No. (%)	2 (6.7)	2 (20.0)	0 (0)	0.103
CRi, No. (%)	6 (20.0)	1 (10.0)	5 (25.0)	0.633
MLFS, No. (%)	11 (36.7)	3 (30.0)	8 (40.0)	0.702
PR, No. (%)	2 (6.7)	0 (0)	2 (10.0)	0.540
NR, No. (%)	9 (30.0)	4 (40.0)	5 (25.0)	
MRD−, No. (%)	9 (42.9)	3 (14.3)	6 (28.6)	
OS				0.184
Median, months (95% CI)	Not reached	Not reached	12.2 (3.0–21.4)	
12-months, % (95% CI)	60.0 (38.0–76.4)	77.8 (36.5–93.9)	50.7 (24.2–72.2)	
EFS				0.816
Median, months (95% CI)	12.2 (NE-NE)	Not reached	12.2 (2.2–22.2)	
12-months, % (95% CI)	54.1 (33.6–70.6)	60.0 (25.3–82.7)	51.1 (26.3–71.4)	

Data are *n* (%) or median (range).

*AML* acute myeloid leukaemia, *WBC* white blood cell count, *ELN* European Leukemia Network, *VEN* venetoclax, *FAB* French-American-British classification systems, *CR* complete remision, *CRi* CR with incomplete hematological recovery, *CRc* CR+CRi, composite complete remission, *MRD* mesurable residual disease, *PR* partial remission, *NR* non-remission, *ORR* CR+CRi+MLFS + PR, overall response rate; *EFS* event-free survival, OS overall survival, *NE* not estimated.

Bold values *P* < 0.05.

Responses to CAV are summarized in Table 1, Supplementary Fig. 2. The ORR rate after the first cycle of CAV was 70% (95% CI 52.1–83.3%). There was no difference in the ORR rate between patients with refractory and relapsed AML (60% vs 75%, *P* = 0.673). Patients with prior venetoclax exposure showed lower ORR compared with those not treated with venetoclax (8/13, 61.5% vs 13/17, 76.5%, *P* = 0.006). 42.9% (9/21) of patients with ORR were MRD negative. 66.7% (6/9) relapsed AML patients who received CAV as the first salvage regimen showed a response (5 CRi, 1 MLFS). All 7 patients with *CEBPA* mutations responded to CAV (1 CR, 1 CRi, 4 MLFS, 1 PR). All 4 patients with *RUNX1* mutations responded (1 CRi, 3 MLFS). 77.8% (7/9) of *NRAS* mutated patients responded to CAV (3 CRi, 3 MLFS, 1 PR). 75% (3/4) of patients with *FLT3*-ITD showed response (1 CRi, 2 MLFS). 75% (3/4) of patients with *FLT3*-TKD responded to CAV (2 CRi, 1 MLFS). 66.7% (4/6) patients with *KMT2A* rearrangements had response (1 CRi, 2 MLFS, 1 PR). Neither of two patients with monosomy 7 or complex karyotype responded to CAV.

Eighteen patients underwent allo-HSCT after CAV (Supplementary Table 3). The median time from completion of CAV to initiate conditioning chemotherapy for transplantation was 17 (1–90) days. At the time of transplantation, 5 patients had active disease, 4 had CR, 1 had CRi and 8 had MLFS. Seventeen patients received a myeloablative conditioning regimen and one patient received reduced intensity conditioning regimen. Of the donors, 5 were matched siblings, 5 were matched/mismatched unrelated, 7 were haploidentical and 1 was a cord blood donor. Nine patients received post-transplant maintenance therapy with azacitidine, and the other nine patients didn’t receive maintenance because of cGVHD or delayed recovery of blood cell counts. Totally, 22.2% (4/18) of patients relapsed after transplantation. Of whom, 3 were CAV nonresponders and relapsed within 3 months post-transplant. One patient achieved CRi after CAV but relapsed at 6 months after transplantation. Three patients received re-induction therapy containing venetoclax, but all did not respond. One patient only received supportive care (Supplementary Fig. 3).

The median follow-up was 12.4 (95% CI, 4.2–20.7) months. Death occurred in 11 patients (Fig. 1a). 58.3% (7/12) of non-transplanted patients died, 4 of disease progression, 2 of pneumonia and 1 of cerebral bleeding. 22.2% (4/18) of transplanted patients died, 3 died of disease progression and 1 of sepsis (Supplementary Fig. 3). The estimated 1-year OS rate was 77.8% (95% CI, 36.5–93.9) and 50.7% (95% CI, 24.2–72.2), in the refractory and relapsed AML group, respectively (Fig. 1b). The estimated 1-year EFS was 60.0% (95% CI, 25.3–82.7) and 51.1% (95% CI, 26.3–71.4), in the refractory and relapsed AML group, respectively (Fig. 1c). Patients with venetoclax exposure had shorter median OS compared with those not treated with venetoclax (Supplementary Fig. 4). Patients who received allo-HSCT had superior OS compared with those who didn’t transplant (Fig. 1d). EFS was comparable in patients with or without allo-HSCT (Fig. 1e). Multivariate COX regression analysis showed that patients with refractory AML (HR = 5.55; 95% CI 1.01–30.45; *P* = 0.048), no prior exposure to venetoclax (HR = 0.11; 95% CI 0.02–0.55; *P* = 0.008) and allo-HSCT (HR = 15.01; 95% CI 2.99–75.52; *P* = 0.001) were associated with prolonged OS (Supplementary Table 4, Supplementary Fig. 5). There was no difference in OS and EFS based on other clinical characteristics (Supplementary Fig. 6).

**Fig. 1:**
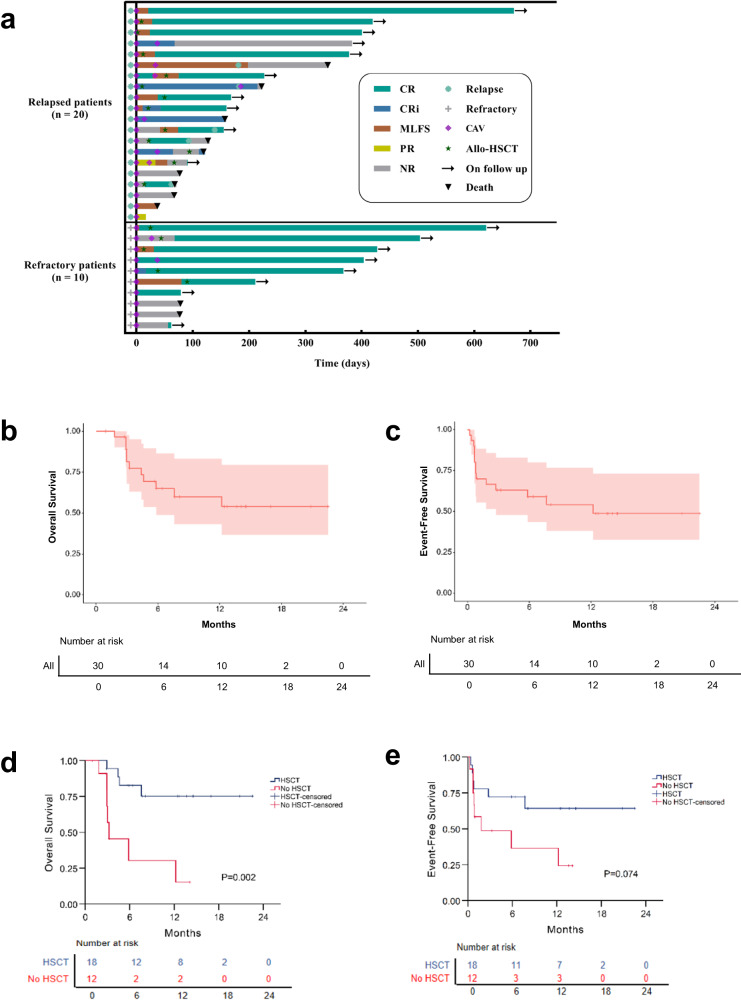
Response outcomes and survival of all the patients. **a** Swimming plot of all the enrolled patients. OS (**b**) and EFS (**c**) of all the patients. OS (**d**) and EFS (**e**) of patients with or without allo-HSCT.

The most common hematologic AEs of grade 3 or higher were neutropenia (76.7%), thrombocytopenia (73.4%), anemia (83.4%) and febrile neutropenia (30.0%). The median time to achieve ANC > 0.5 × 10^9^/L and PLT > 20 × 10^9^/L in the responded patients were 21 (range, 3–28) and 17 (range, 8–21) days, respectively. In the 18 transplanted patients, the median time to neutrophils and platelets engraftment were 13.5 (range, 10–20) and 14.5 (range, 8–43) days, respectively. The most common nonhematologic AEs of grade 3 or higher were pneumonia (16.7%), other infections (10%) and sepsis (6.7%). No patients experienced tumor lysis syndrome (Supplementary Table 5).

Regimens containing venetoclax have been studied in R/R AML. A regimen comprised of homoharringtonine, venetoclax and azacitidine (VAH) showed a CRc of 70.8% and MRD negativity of 58.8% for R/R AML [12]. The addition of venetoclax to fludarabine, idarubicin (FLAVIDA) demonstrated an ORR of 78% in another prospective study [13]. These data suggest that venetoclax may have synergistic anti-leukemia effects with drugs such as homoharringtonine and fludarabine. In our study, CAV demonstrated an ORR of 70% in the overall patients, showing similar efficacy as compared with the VAH and FLAVIDA regimen. Regarding the treatment-related AEs, the incidence of grade 3–4 febrile neutropenia and sepsis were 37.4% and 11.4%, respectively for the VAH regimen [12]. And the incidence of febrile neutropenia and bacteraemia/sepsis was 77 and 23% for the FLAVIDA regimen [13]. In this study, the incidence of febrile neutropenia and sepsis were 30% and 6.7%, respectively, which was both lower than VAH and FLAVIDA. Therefore, our data suggest that CAV has comparable efficacy and a better safety profile compared to the salvage regimens described above.

All patients with *CEBPA* or *RUNX1* mutations responded to CAV, suggesting that these patients may be sensitive to CAV. *RAS* mutations are found in about 30% of AML patients and were considered as late events in AML. *N/KRAS* mutated LSCs altered BCL2 family expression profile and conferred clinical resistance to venetoclax [14]. 77.8% of patients with *NRAS* mutations in this study responded to CAV, which demonstrated that CLAD might overcome the resistance to venetoclax in *NRAS* mutated AML patients and provided evidence for the application of CAV in *NRAS* mutated AML patients. In a phase 2 trial, the venetoclax added to alternating CLAD plus LDAC and azacitidine regimen achieved remission rates of 57% in patients with ND AML having *TP53* mutations/loss, as well as high rates of MRD negativity (63%) in responders [15]. Since there were no *TP53* mutated patients in this study, the efficacy of CAV in *TP53* mutated R/R AML warrants further study with an expanded sample.

In summary, this is the first study to explore the efficacy and safety of the CAV regimen bridging to allo-HSCT for R/R AML, with high response rates and encouraging survival. Because this was a single-arm study and the sample size was small, the value of the CAV regimen compared with conventional chemotherapy in R/R AML is currently underway in a randomized controlled study (NCT05657639).

### Supplementary information

Supplementary Material

## Data Availability

The data that support the findings of this study are available from the corresponding author upon reasonable request.
