# Supplementary material for: Efficacy and safety of cladribine, low-dose cytarabine and venetoclax in relapsed/refractory acute myeloid leukemia: results of a pilot study
Source: Blood Cancer J. 2024 Jan 18;14(1):12. doi: 10.1038/s41408-024-00982-3 (PMC10796351; doi:10.1038/s41408-024-00982-3)

Efficacy and safety of cladribine, low-dose cytarabine and venetoclax in relapsed/refractory acute myeloid leukemia：results of a pilot study

Yan-Yan Li[1,2†], Shuai-Shuai Ge[1,2†], Yuan-Hong Huang[1,2†], Ming-Zhu Xu[1,2†], Chao-Ling Wan[1,2], Kai-Wen Tan[1,2], Tao Tao[3], Hai-Xia Zhou*[1,2], Sheng-Li Xue*[1,2], Hai-Ping Dai*[1,2]

Statistical methods

The cutoff date of clinical data was April 10, 2023. Descriptive analyses of patient characteristics included median and range for continuous variables, absolute and relative frequencies for categorical variables. Comparisons of categorical variables between groups were performed using the Chi-squared test or Fisher's exact test, and comparisons of continuous variables were performed using the Mann-Whitney U test. Probabilities of OS and EFS were calculated by using the Kaplan-Meier method. Differences between OS and EFS were analyzed using the log-rank test. Cox regression models were used to analyze the correlation of OS with the variables. Variables that were significantly associated with prognosis or had a P value < 0.05 were included in the multivariate analysis. P values < 0.05 were considered to be statistically significant. SPSS, version 26.0 (Illinois, USA) and R, version 4.0.0 were used for statistical analyses.

Brief study protocol

Title: CAV regimen bridging to HSCT for R/R acute leukemia (NCT05190549)

Brief summary

Although allogeneic hematopoietic stem cell transplantation (HSCT) is a curative treatment option for acute leukemia (AL), relapsed or refractory (R/R) AL is still a big challenge. It is believed that decreased tumor burden before HSCT is a favorable factor contributing to the long-term survival of R/R AL patients and many kinds of bridging chemotherapy regimens were devised to kill leukemic cells before HSCT, there is still no consensus that which regimen is optimal. This study is to investigate the curative efficacy and safety of bridging CAV (cladribine combined with low dose Ara-C and venetoclax) regimens followed by HSCT treatment protocol for R/R AML.

Eligibility Criteria

Inclusion Criteria:

1. Patients with AL must meet one of the following criteria:

A or B. A: relapsed AL disease was defined as follows: (1) Reappearance of leukemic blasts in the peripheral blood after CR; or (2) detection of >5% blasts in the bone marrow (BM) not attributable to another cause(e.g. regeneration after consolidation therapy); or (3) extramedullary relapse. B: refractory AL disease was defined as follows: (1) failure to achieve CR, CRh or CRi after two courses of intensive induction treatment. or (2) failure to achieve complete remission after one cycle of induction chemotherapy or a reduction in the number of blasts of less than 50% after one cycle of induction treatment, with residual blasts >15%.

2. Patients without ≥grade 3 of cardiac, hepatic, pulmonary, or renal dysfunctions.
3. Sign informed consent.
4. Patients aged between 16 and 70 years old, male or female is permitted.
5. ECOG performance status score less than 1.
6. The expected survival is longer than 3 months.

Exclusion Criteria:

1. With other malignant tumors.
2. Patients received the treatment of cladribine or venetoclax.
3. Patients received cardiac angioplasty or stent implantation.
4. Active infections that are not under clinical control (bacteria or fungus or virus is included).
5. Liver functions abnormalities (total bilirubin>1.5 times the upper limit of the normal range, ALT/AST>2.5 times the upper limit of the normal range or patients with liver involvement whose ALT/AST>1.5 times the upper limit of the normal range), or renal anomalies (serum creatinine>1.5 times the upper limit of normal value).
6. Pregnant or nursing women.
7. Patients with mental disorders or other conditions whereby informed consent cannot be obtained and where the requirements of the study treatment and procedures cannot be met.
8. Patients participated in any other clinical trials 3 months prior to signing the informed consent.
9. Patients not suitable for the study according to the investigator's assessment.

Primary endpoint

ORR (overall response rate): calculated as the sum of CR, CRi, MLFS and PR after the completion of the CAV regimen.

Secondary endpoint

1. Overall survival (OS): defined as the time from enrollment to this study to the date of death from any cause; patients not known to have died at last follow-up are censored on the date they were last known to be alive.
2. Event-free survival (EFS): defined as the time from the initiation of CAV to treatment failure, relapse, death from any cause or the last follow-up.
3. AEs (Adverse events): evaluated and graded according to CTCAE 5.0.

SUPPLEMENTARY TABLES

SUPPLEMENTARY TABLE 1. Treatments prior to CAV regimen.

Pt No	Sex	Age (Year)	Treatments prior to CAV	Treatment lines before CAV
1	Male	39	IA, HA, DA, DAE, FLAG, DAC+HAG+VEN, AZA+VEN	4
2	Female	39	IAC, MD-Ara-C+IDA	2
3	Female	34	DAC+HAAG	1
4	Female	41	DAC+HAAG, IA	2
5	Female	29	IA, ID- Ara-C, DAC+HAAG	2
6	Female	58	IA, CLAG	2
7	Female	53	DAC+VEN, ID-Ara-C, CLAG	2
8	Male	35	IA, FLAG	2
9	Male	55	IA	1
10	Female	17	IA, ID-Ara-C+IDA, ID-Ara-C+DAC	2
11	Male	68	AZA+VEN, AZA+VEN+CHD	2
12	Female	62	AZA+VEN, AZA+VEN+CHD	2
13	Male	16	IA, IA+ sorafenib, ID-Ara-C+DAC	2
14	Male	58	AZA+HAAG, HD-Ara-C, ASCT	2
15	Female	22	IA, Ara-C+IDA	2
16	Male	38	IA, ID-Ara-C+CLD, IA+DAC, ID-Ara-C+IDA	3
17	Male	32	IA, AZA+VEN	2
18	Female	27	DAC+VEN, ID-Ara-C+IDA	1
19	Male	61	CPX351	1
20	Male	37	HAG, IDA, IA, ID-Ara-C+IDA, AZA+VEN+CHD	3
21	Female	47	HAG, CLAG	2
22	Male	37	IA, ID-Ara-C+IDA	1
23	Female	40	DAC+HAAG, ID-Ara-C+IDA	1
24	Male	58	DAC+VEN, ID-Ara-C	2
25	Male	59	AZA+VEN+CHD, ID-Ara-C+IDA	1
26	Female	32	IA, DAC+EA, CLAG	2
27	Male	67	AZA+VEN+MTN, MA	1
28	Male	54	DAC+VEN, ID-Ara-C+IDA	1
29	Male	29	DAC+VEN	1
30	Female	58	DAC+VEN	1

Abbreviations: ASCT, autologous hematopoietic stem cell transplantation; allo-HSCT, allogeneic hematopoietic stem cell transplantation; AZA, azacitidine; CHD, chidamide; CLAG, cladribine, cytarabine and granulocyte colony stimulating factor; CLD, cladribine; CPX351, vyxeos; DA, daunorubicin and cytarabine; DAC, decitabine; DAE, daunorubicin, cytarabine and etoposide; FLAG, fludarabine, cytarabine and granulocyte colony stimulating factor; HA, homoharringtonine and cytarabine; HAG, homoharringtonine, cytarabine and granulocyte colony stimulating

factor; HAAG, homoharringtonine, cytarabine, aclarubicin, and granulocyte colony stimulating factor; IA, idarubicin and cytarabine; IAC, idarubicin, cytarabine and cladribine; IDA, idarubicin; ID-Ara-C, intermediate-dose cytarabine; MA, mitoxantrone and cytarabine; MTN: mitoxantrone; VEN, venetoclax.

SUPPLEMENTARY TABLE 2. Treatment regimens for responders post the CAV regime.

Pt No	Sex	Age (Year)	Treatment regimens after CAV
2	Female	39	allo-HSCT
3	Female	34	allo-HSCT
5	Female	29	CAV consolidation for two courses
6	Female	58	allo-HSCT
7	Female	53	allo-HSCT
9	Male	55	CAV, ID-Ara-C, IA
10	Female	17	allo-HSCT
12	Female	62	CAV consolidation for two courses until relapse
14	Male	58	allo-HSCT
15	Female	22	allo-HSCT
16	Male	38	CAV/MA for consolidation until relapse
17	Male	32	CAV consolidation once and allo-HSCT
18	Female	27	CAV consolidation once and allo-HSCT
20	Male	37	supportive care
21	Female	47	allo-HSCT
22	Male	37	allo-HSCT
23	Female	40	allo-HSCT
24	Male	58	supportive care
25	Male	59	CAV, VEN+gilteritinib, allo-HSCT
28	Male	54	CAV, allo-HSCT
30	Female	58	MA

Abbreviations: allo-HSCT, allogeneic hematopoietic stem cell transplantation; CAV, cladribine, cytarabine and venetoclax; HD-Ara-C, high- dose cytarabine; IA, idarubicin and cytarabine; MA, mitoxantrone and cytarabine.

SUPPLEMENTARY TABLE 3. Characteristics for patients receiving allo-HSCT.

Characteristics	Total
**No.**	18
Median age, years (range)	39 (16-59)
Gender, male, no. (%)	8 (44.4)
Median duration from completion of CAV to allo-HSCT (range)	17 (1-90)
Disease status prior to allo-HSCT, no. (%)	
CR	4 (22.2)
CRi	1 (5.6)
MLFS	8 (44.4)
NR	5 (27.8)
Donor source, (no.%)	
Haplo-d	7 (38.9)
MUD	3 (16.7)
MMUD	2 (11.1)
MSD	5 (27.8)
CBT	1 (5.6)
Donor-recipient	
Female to male	3 (16.7)
others	15 (83.3)
Median CD34 cells, $\times10^6$/kg (range)	3.17 (0.82-9.03)
Median CD3 cells, $\times10^8$/kg (range)	1.83 (0.95-3.27)
Neutrophil engraftment, days (range)	13.5 (10-20)
Platelet engraftment, days (range)	14.5 (8-43)
aGVHD	4 (22.2)
Grade 1-2	4 (22.2)
Grade 3-4	0 (0)
cGVHD	8 (44.4)
Limited	3 (16.7)
Extensive	5 (27.8)
Relapse after allo-HSCT, no. (%)	4 (22.2)
Median follow-up time in survivor, months (range)	12.7 (2.9-22.5)
Estimated 1-year OS, % (95% CI)	75.1 (46.0-90.0)
Estimated 1-year EFS, % (95% CI)	64.2 (36.2-84.4)

Abbreviations: Allo-HSCT, allogeneic hematopoietic stem cell transplantation; HLA, human leukocyte antigen; PBSC, peripheral blood stem cell; NC, Nucleated cells; CR, complete remission; CRi, complete remission with incomplete hematological recovery.

SUPPLEMENTARY TABLE 4. Multivariable Cox Model for OS.

	Multivariable Model	
**Covariates**	**HR（95% CI）**	**P value**
AML status (relapsed vs. refractory)	5.55 (1.01-30.45)	**0.048**
Prior exposure to VEN (No vs. Yes)	0.11 (0.02-0.55)	**0.008**
Allo-HSCT (No vs. Yes)	15.01 (2.99-75.52)	**0.001**

Abbreviations: OS, overall survival; HR, Hazard Ratio; CI, confidence interval; VEN, Venetoclax; HSCT, Hematopoietic stem cell transplantation.

SUPPLEMENTARY TABLE 5. Adverse events during the CAV treatment.

Event	Total	Grade 1	Grade 2	Grade 3	Grade 4
Adverse events (n=30)					
Hematologic AEs					
Febrile neutropenia	9 (30.0)	0	0	8 (26.7)	1 (3.3)
Anemia	29 (96.7)	1 (3.3)	3 (10.0)	20 (66.7)	5 (16.7)
Thrombocytopenia	28 (93.3)	0	6 (20.0)	8 (26.7)	14 (46.7)
Neutropenia	26 (86.7)	0	3 (10.0)	2 (6.7)	21 (70.0)
GI AEs					
Nausea	8 (26.7)	1 (3.3)	7 (23.3)	0	0
Vomiting	3 (10.0)	3 (10.0)	0	0	0
Diarrhea	2 (6.7)	1 (3.3)	1 (3.3)	0	0
Constipation	1 (3.3)	1 (3.3)	0	0	0
Elevated ALT or AST	5 (16.7)	5 (16.7)	0	0	0
Infections					
Pneumonia	5 (16.7)	0	0	5 (16.7)	0
Sepsis	2 (6.7)	0	0	2 (6.7)	0
Other infections	4 (13.4)	0	1 (3.3)	3 (10.0)	0
Other AEs					
Hypokalemia	3 (10.0)	2 (6.7)	1 (3.3)	0	0
Fatigue	7 (23.3)	3 (10.0)	4 (13.3)	0	0
Pruritis	3 (10.0)	1 (3.3)	2 (6.7)	0	0
Epistaxis	1 (3.3)	0	1 (3.3)	0	0
Cough	1 (3.3)	1 (3.3)	0	0	0

Supplementary Figures

Supplementary Figure legends

Supplementary Figure 1. Mutational landscapes of 30 patients with refractory /relapsed AML. Each column represents a single patient. CR, complete remission; CRi, CR with incomplete hematological recovery; MLFS, morphologic leukemia-free state; PR, partial remission; NR, non-remission; MRD, measurable residual disease; Risk, 2017 ELN risk stratification.

Supplementary Figure 2. Treatment response to CAV of all the patients.

Supplementary Figure 3. Flowchart for the enrollment and treatment of all the patients.

Supplementary Figure 4. OS and EFS of patients with or without history of VEN exposure.

Supplementary Figure 5. Forest plot of HRs (95%CI) derived multivariate Cox models for OS.

Supplementary Figure 6. Survival of the patients based on other clinical characteristics. (a)(e) OS and EFS of patients with Lower or higher WBC. (b)(f) OS and EFS of patients with blast > 50% or ≤ 50%. (c)(g) OS and EFS of patients with FAB M4/5 subtype or other subtypes. (d)(h) OS and EFS of patients with different risk stratifications according to 2017 ELN guidelines.

Supplementary Figure 1

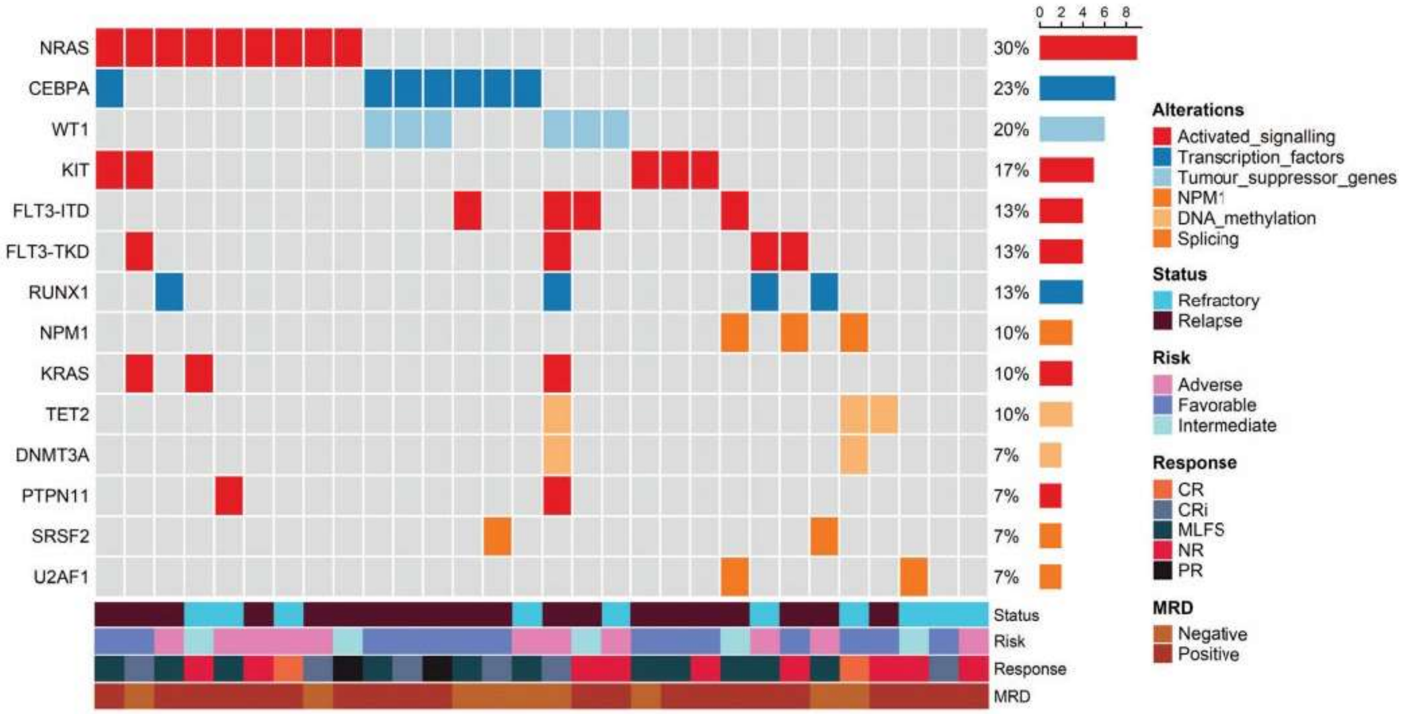

Supplementary Figure 2

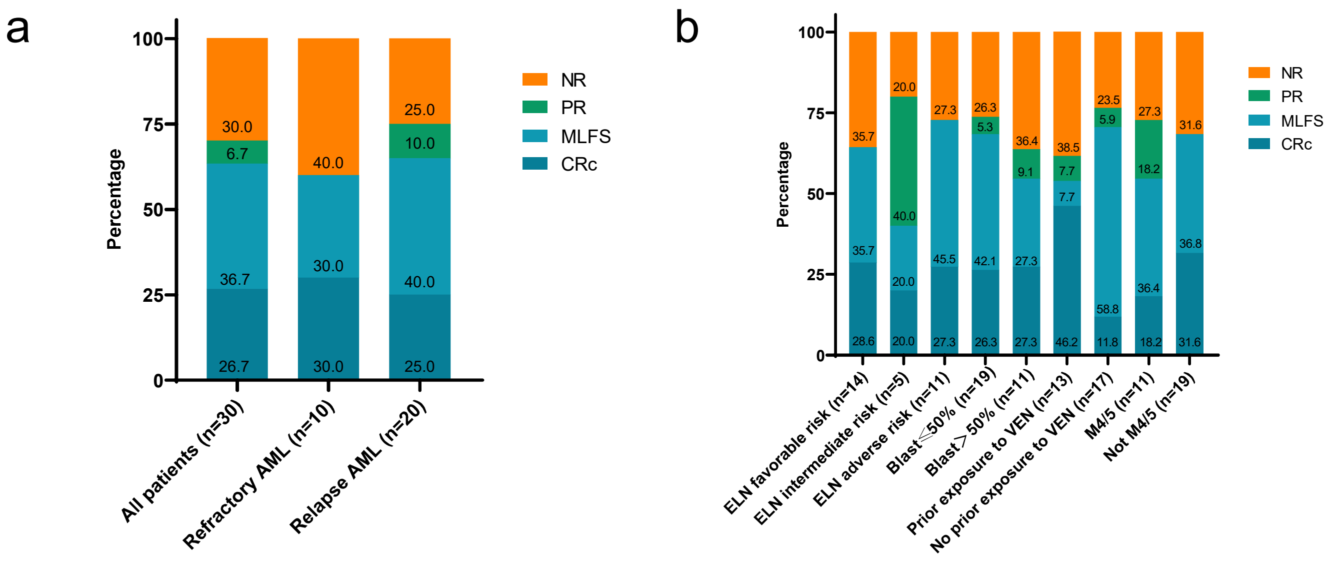

Supplementary Figure 3

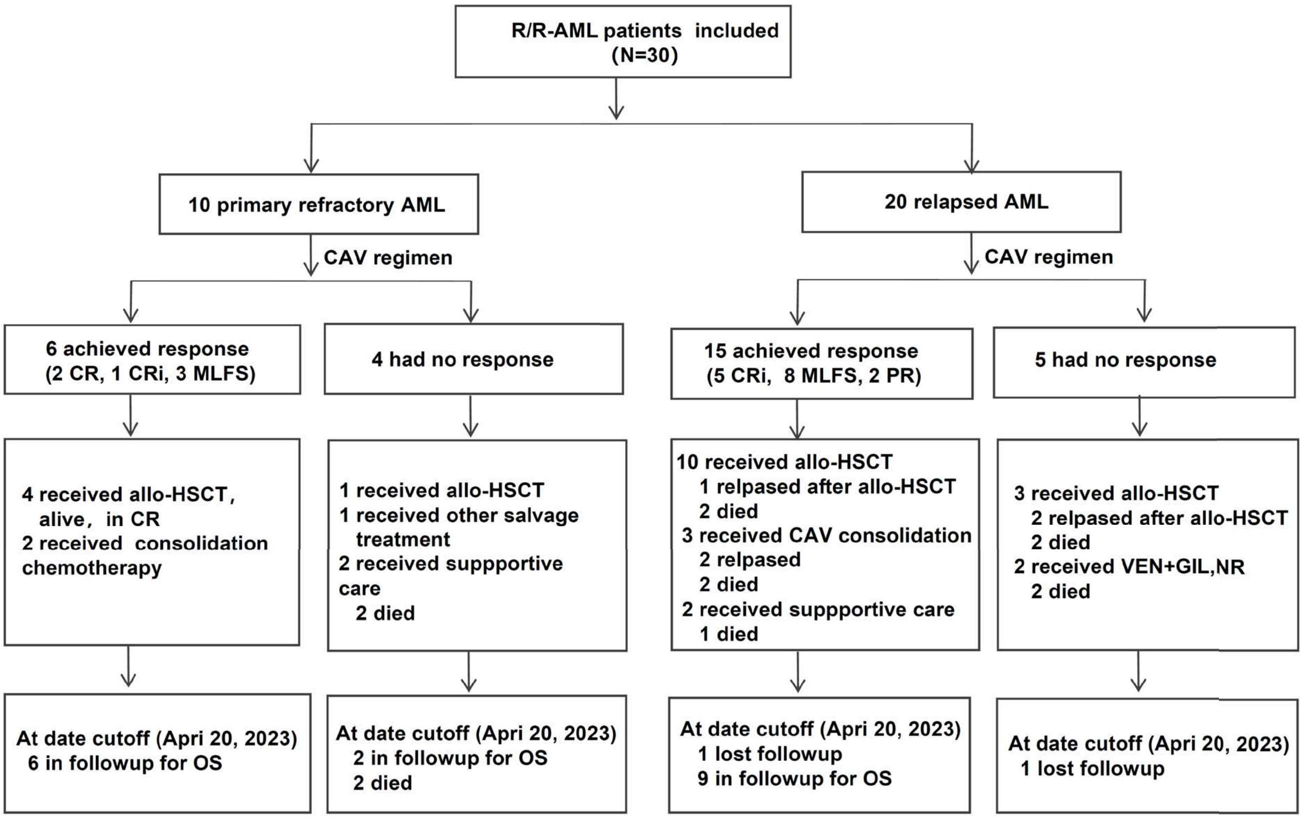

Supplementary Figure 4

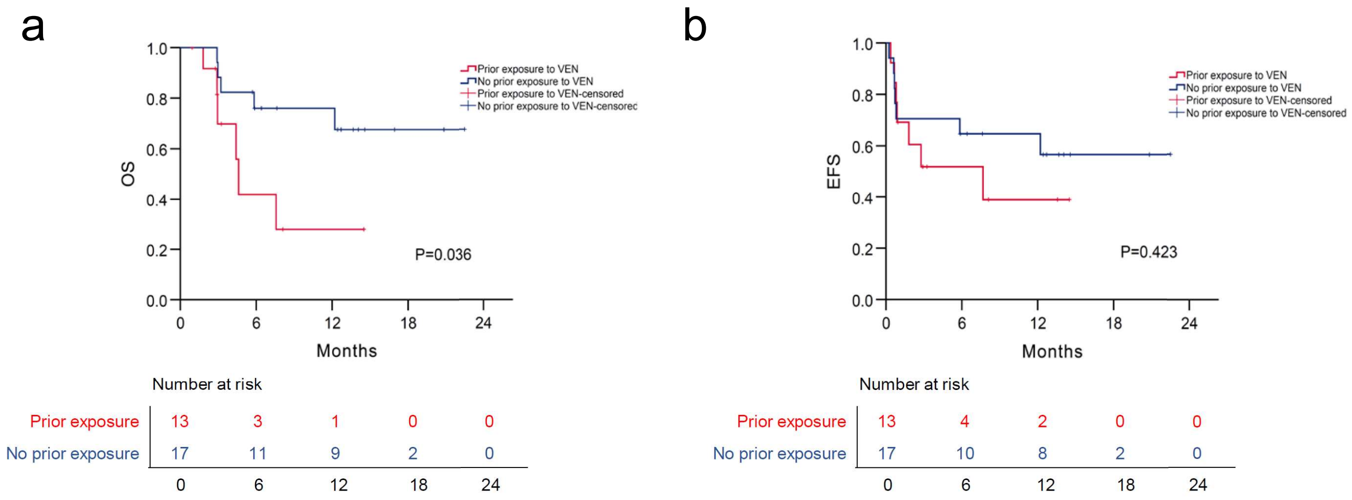

Supplementary Figure 5

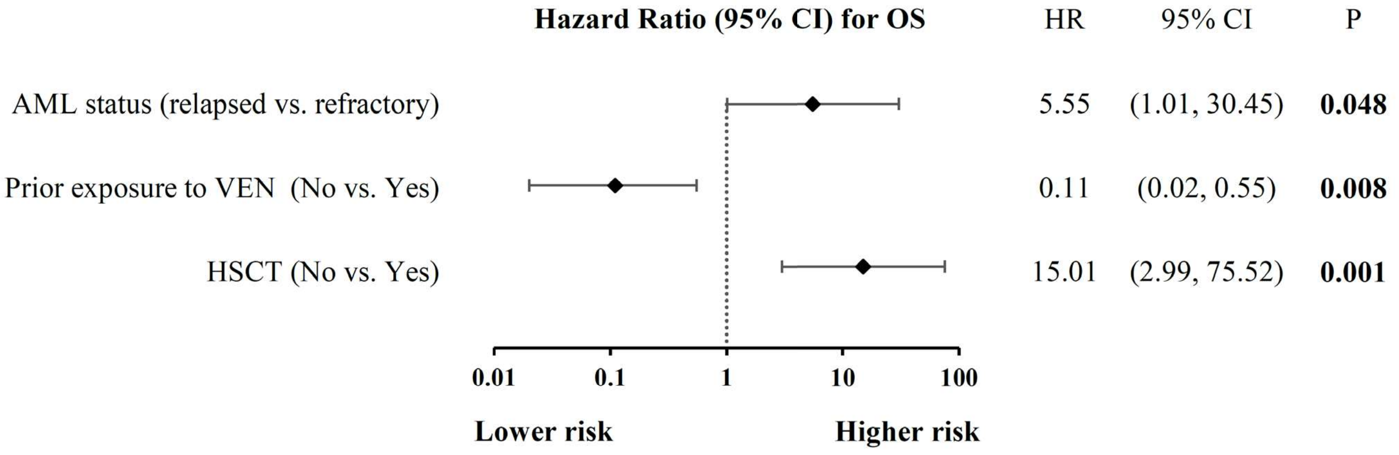

Supplementary Figure 6

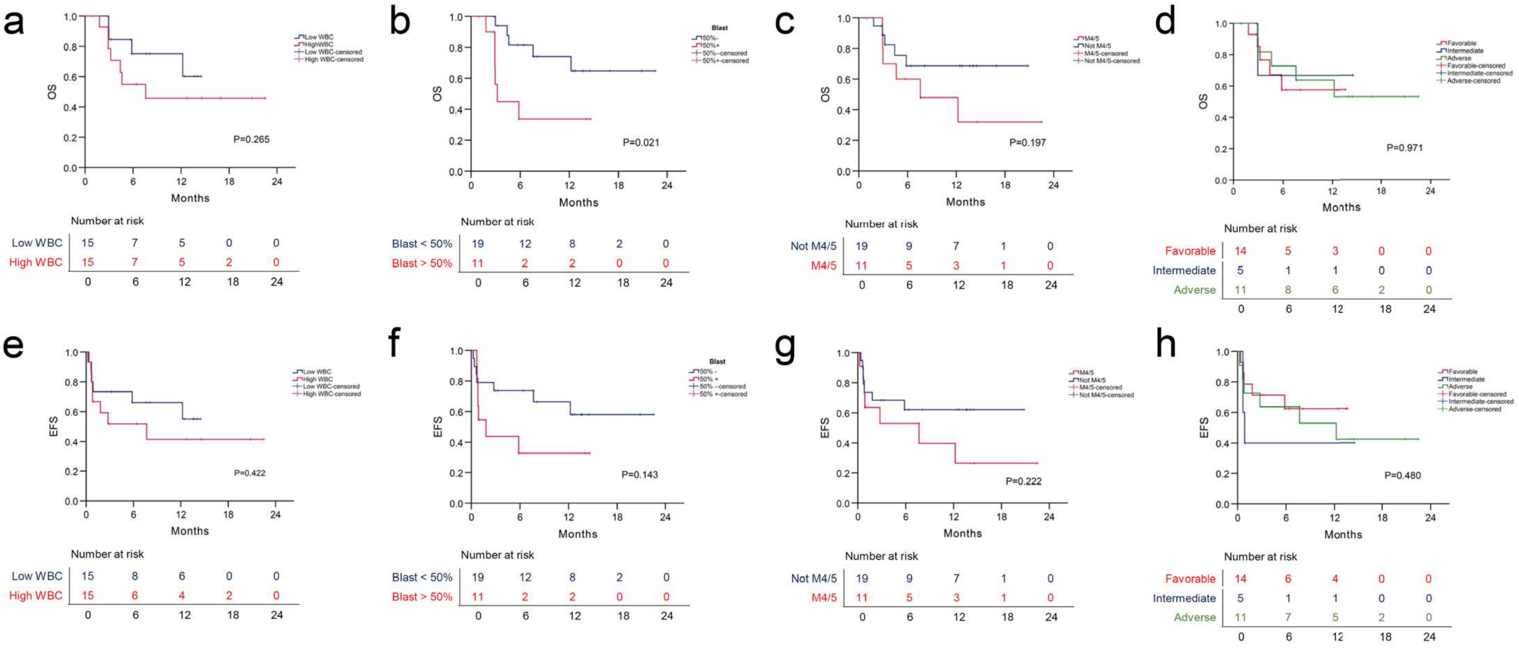

Supplement: Supplementary file 1 — Supplementary Material [file 41408_2024_982_MOESM1_ESM.pdf]
